# The Implementation Research Logic Model: a method for planning, executing, reporting, and synthesizing implementation projects

**DOI:** 10.1186/s13012-020-01041-8

**Published:** 2020-09-25

**Authors:** Justin D. Smith, Dennis H. Li, Miriam R. Rafferty

**Affiliations:** 1 Department of Population Health Sciences, University of Utah School of Medicine, Salt Lake City, Utah USA; 2 Center for Prevention Implementation Methodology for Drug Abuse and HIV, Department of Psychiatry and Behavioral Sciences, Department of Preventive Medicine, Department of Medical Social Sciences, and Department of Pediatrics, Northwestern University Feinberg School of Medicine, Chicago, Illinois USA; 3 Center for Prevention Implementation Methodology for Drug Abuse and HIV, Department of Psychiatry and Behavioral Sciences, Feinberg School of Medicine; Institute for Sexual and Gender Minority Health and Wellbeing, Northwestern University Chicago, Chicago, Illinois USA; 4 Shirley Ryan AbilityLab and Center for Prevention Implementation Methodology for Drug Abuse and HIV, Department of Psychiatry and Behavioral Sciences and Department of Physical Medicine and Rehabilitation, Northwestern University Feinberg School of Medicine, Chicago, Illinois USA

**Keywords:** Logic models, Program theory, Integration, Study specification

## Abstract

**Background:**

Numerous models, frameworks, and theories exist for specific aspects of implementation research, including for determinants, strategies, and outcomes. However, implementation research projects often fail to provide a coherent rationale or justification for how these aspects are selected and tested in relation to one another. Despite this need to better specify the conceptual linkages between the core elements involved in projects, few tools or methods have been developed to aid in this task. The Implementation Research Logic Model (IRLM) was created for this purpose and to enhance the rigor and transparency of describing the often-complex processes of improving the adoption of evidence-based interventions in healthcare delivery systems.

**Methods:**

The IRLM structure and guiding principles were developed through a series of preliminary activities with multiple investigators representing diverse implementation research projects in terms of contexts, research designs, and implementation strategies being evaluated. The utility of the IRLM was evaluated in the course of a 2-day training to over 130 implementation researchers and healthcare delivery system partners.

**Results:**

Preliminary work with the IRLM produced a core structure and multiple variations for common implementation research designs and situations, as well as guiding principles and suggestions for use. Results of the survey indicated a high utility of the IRLM for multiple purposes, such as improving rigor and reproducibility of projects; serving as a “roadmap” for how the project is to be carried out; clearly reporting and specifying how the project is to be conducted; and understanding the connections between determinants, strategies, mechanisms, and outcomes for their project.

**Conclusions:**

The IRLM is a semi-structured, principle-guided tool designed to improve the specification, rigor, reproducibility, and testable causal pathways involved in implementation research projects. The IRLM can also aid implementation researchers and implementation partners in the planning and execution of practice change initiatives. Adaptation and refinement of the IRLM are ongoing, as is the development of resources for use and applications to diverse projects, to address the challenges of this complex scientific field.

Contributions to the literature

- Drawing from and integrating existing frameworks, models, and theories, the IRLM advances the traditional logic model for the requirements of implementation research and practice.
- The IRLM provides a means of describing the complex relationships between critical elements of implementation research and practice in a way that can be used to improve the rigor and reproducibility of research and implementation practice, and the testing of theory.
- The IRLM offers researchers and partners a useful tool for the purposes of planning, executing, reporting, and synthesizing processes and findings across the stages of implementation projects.

## Background

In response to a call for addressing noted problems with transparency, rigor, openness, and reproducibility in biomedical research [1], the National Institutes of Health issued guidance in 2014 pertaining to the research it funds (https://www.nih.gov/research-training/rigor-reproducibility). The field of implementation science has similarly recognized a need for better specification with similar intent [2]. However, integrating the necessary conceptual elements of implementation research, which often involves multiple models, frameworks, and theories, is an ongoing challenge. A conceptually grounded organizational tool could improve rigor and reproducibility of implementation research while offering additional utility for the field.

This article describes the development and application of the Implementation Research Logic Model (IRLM). The IRLM can be used with various types of implementation studies and at various stages of research, from planning and executing to reporting and synthesizing implementation studies. Example IRLMs are provided for various common study designs and scenarios, including hybrid designs and studies involving multiple service delivery systems [3, 4]. Last, we describe the preliminary use of the IRLM and provide results from a post-training evaluation. An earlier version of this work was presented at the 2018 AcademyHealth/NIH Conference on the Science of Dissemination and Implementation in Health, and the abstract appeared in the *Implementation Science* [5].

### Specification challenges in implementation research

Having an imprecise understanding of what was done and why during the implementation of a new innovation obfuscates identifying the factors responsible for successful implementation and prevents learning from what contributed to failed implementation. Thus, improving the specification of phenomena in implementation research is necessary to inform our understanding of how implementation strategies work, for whom, under what determinant conditions, and on what implementation and clinical outcomes. One challenge is that implementation science uses numerous models and frameworks (hereafter, “frameworks”) to describe, organize, and aid in understanding the complexity of changing practice patterns and integrating evidence-based health interventions across systems [6]. These frameworks typically address implementation determinants, implementation process, or implementation evaluation [7]. Although many frameworks incorporate two or more of these broad purposes, researchers often find it necessary to use more than one to describe the various aspects of an implementation research study. The conceptual connections and relationships between multiple frameworks are often difficult to describe and to link to theory [8].

Similarly, reporting guidelines exist for some of these implementation research components, such as strategies [9] and outcomes [10], as well as for entire studies (i.e., Standards for Reporting Implementation Studies [11]); however, they generally help describe the individual components and not their interactions. To facilitate causal modeling [12], which can be used to elucidate mechanisms of change and the processes involved in both successful and unsuccessful implementation research projects, investigators must clearly define the relations among variables in ways that are testable with research studies [13]. Only then can we open the “black box” of how specific implementation strategies operate to predict outcomes.

### Logic models

Logic models, graphic depictions that present the shared relationships among various elements of a program or study, have been used for decades in program development and evaluation [14] and are often required by funding agencies when proposing studies involving implementation [15]. Used to develop agreement among diverse stakeholders of the “what” and the “how” of proposed and ongoing projects, logic models have been shown to improve planning by highlighting theoretical and practical gaps, support the development of meaningful process indicators for tracking, and aid in both reproducing successful studies and identifying failures of unsuccessful studies [16]. They are also useful at other stages of research and for program implementation, such as organizing a project/grant application/study protocol, presenting findings from a completed project, and synthesizing the findings of multiple projects [17].

Logic models can also be used in the context of program theory, an explicit statement of how a project/strategy/intervention/program/policy is understood to contribute to a chain of intermediate results that eventually produce the intended/observed impacts [18]. Program theory specifies both a Theory of Change (i.e., the central processes or drivers by which change comes about following a formal theory or tacit understanding) and a Theory of Action (i.e., how program components are constructed to activate the Theory of Change) [16]. Inherent within program theory is causal chain modeling. In implementation research, Fernandez et al. [19] applied mapping methods to implementation strategies to postulate the ways in which changes to the system affect downstream implementation and clinical outcomes. Their work presents an implementation mapping logic model based on Proctor et al. [20, 21], which is focused primarily on the selection of implementation strategy(s) rather than a complete depiction of the conceptual model linking all implementation research elements (i.e., determinants, strategies, mechanisms of action, implementation outcomes, clinical outcomes) in the detailed manner we describe in this article.

### Development of the IRLM

The IRLM began out of a recognition that implementation research presents some unique challenges due to the field’s distinct and still codifying terminology [22] and its use of implementation-specific and non-specific (borrowed from other fields) theories, models, and frameworks [7]. The development of the IRLM occurred through a series of case applications. This began with a collaboration between investigators at Northwestern University and the Shirley Ryan AbilityLab in which the IRLM was used to study the implementation of a new model of patient care in a new hospital and in other related projects [23]. Next, the IRLM was used with three already-funded implementation research projects to plan for and describe the prospective aspects of the trials, as well as with an ongoing randomized roll-out implementation trial of the Collaborative Care Model for depression management [Smith JD, Fu E, Carroll AJ, Rado J, Rosenthal LJ, Atlas JA, Burnett-Zeigler I, Carlo, A, Jordan N, Brown CH, Csernansky J: Collaborative care for depression management in primary care: a randomized rollout trial using a type 2 hybrid effectiveness-implementation design submitted for publication]. It was also applied in the later stages of a nearly completed implementation research project of a family-based obesity management intervention in pediatric primary care to describe what had occurred over the course of the 3-year trial [24]. Last, the IRLM was used as a training tool in a 2-day training with 63 grantees of NIH-funded planning project grants funded as part of the Ending the HIV Epidemic initiative [25]. Results from a survey of the participants in the training are reported in the “Results” section. From these preliminary activities, we identified a number of ways that the IRLM could be used, described in the section on “Using the IRLM for different purposes and stages of research.”

## Methods

### The Implementation Research Logic Model

#### Structure

In developing the IRLM, we began with the common “pipeline” logic model format used by AHRQ, CDC, NIH, PCORI, and others [16]. This structure was chosen due to its familiarity with funders, investigators, readers, and reviewers. Although a number of characteristics of the pipeline logic model can be applied to implementation research studies, there is an overall misfit due to implementation research’s focusing on the systems that support adoption and delivery of health practices; involving multiple levels within one or more systems; and having its own unique terminology and frameworks [3, 22, 26]. We adapted the typical evaluation logic model to integrate existing implementation science frameworks as its core elements while keeping to the same aim of facilitating causal modeling.

**Fig. 1:**
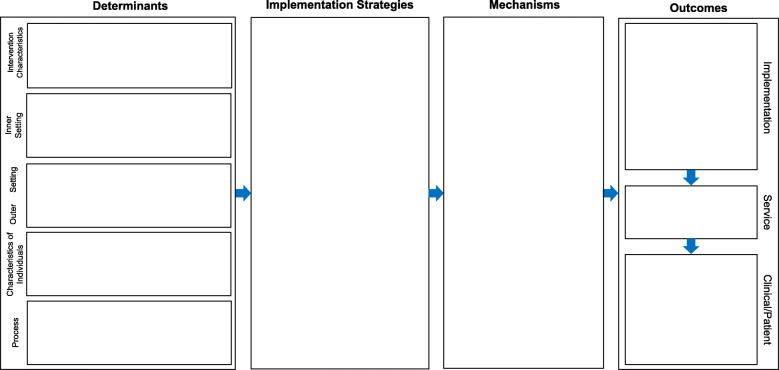
Implementation Research Logic Model (IRLM) Standard Form. *Notes.* Domain names in the determinants section were drawn from the Consolidated Framework for Implementation Research. The format of the outcomes column is from Proctor et al. 2011

**Fig. 2:**
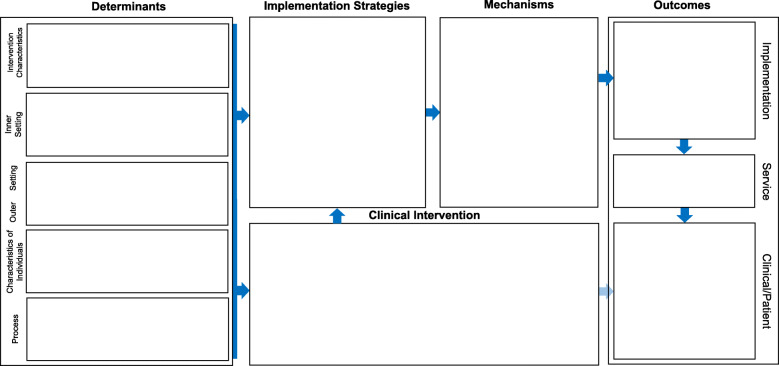
Implementation Research Logic Model (IRLM) Standard Form with Intervention. *Notes.* Domain names in the determinants section were drawn from the Consolidated Framework for Implementation Research. The format of the outcomes column is from Proctor et al. 2011

The most common IRLM format is depicted in Fig. 1. Additional File A1 is a Fillable PDF version of Fig. 1. In certain situations, it might be preferable to include the evidence-based intervention (EBI; defined as a clinical, preventive, or educational protocol or a policy, principle, or practice whose effects are supported by research [27]) (Fig. 2) to demonstrate alignment of contextual factors (determinants) and strategies with the components and characteristics of the clinical intervention/policy/program and to disentangle it from the implementation strategies. Foremost in these indications are “home-grown” interventions, whose components and theory of change may not have been previously described, and novel interventions that are early in the translational pipeline, which may require greater detail for the reader/reviewer. Variant formats are provided as Additional Files A2 to A4 for use with situations and study designs commonly encountered in implementation research, including comparative implementation studies (A2), studies involving multiple service contexts (A3), and implementation optimization designs (A4). Further, three illustrative IRLMs are provided, with brief descriptions of the projects and the utility of the IRLM (A5, A6 and A7).

#### Core elements and theory

The IRLM specifies the relationships between determinants of implementation, implementation strategies, the mechanisms of action resulting from the strategies, and the implementation and clinical outcomes affected. These core elements are germane to every implementation research project in some way. Accordingly, the generalized theory of the IRLM posits that (1) implementation strategies selected for a given EBI are related to implementation determinants (context-specific barriers and facilitators), (2) strategies work through specific mechanisms of action to change the context or the behaviors of those within the context, and (3) implementation outcomes are the proximal impacts of the strategy and its mechanisms, which then relate to the clinical outcomes of the EBI. Articulated in part by others [9, 12, 21, 28, 29], this causal pathway theory is largely explanatory and details the Theory of Change and the Theory of Action of the implementation strategies in a single model. The EBI Theory of Action can also be displayed within a modified IRLM (see Additional File A**4**). We now briefly describe the core elements and discuss conceptual challenges in how they relate to one another and to the overall goals of implementation research.

#### Determinants

Determinants of implementation are factors that might prevent or enable implementation (i.e., barriers and facilitators). Determinants may act as moderators, “effect modifiers,” or mediators, thus indicating that they are links in a chain of causal mechanisms [12]. Common determinant frameworks are the Consolidated Framework for Implementation Research (CFIR) [30] and the Theoretical Domains Framework [31].

#### Implementation strategies

Implementation strategies are supports, changes to, and interventions on the system to increase adoption of EBIs into usual care [32]. Consideration of determinants is commonly used when selecting and tailoring implementation strategies [28, 29, 33]. Providing the theoretical or conceptual reasoning for strategy selection is recommended [9]. The IRLM can be used to specify the proposed relationships between strategies and the other elements (determinants, mechanisms, and outcomes) and assists with considering, planning, and reporting all strategies in place during an implementation research project that could contribute to the outcomes and resulting changes

Because implementation research occurs within dynamic delivery systems with multiple factors that determine success or failure, the field has experienced challenges identifying consistent links between individual barriers and specific strategies to overcome them. For example, the Expert Recommendations for Implementing Change (ERIC) compilation of strategies [32] was used to determine which strategies would best address contextual barriers identified by CFIR [29]. An online CFIR–ERIC matching process completed by implementation researchers and practitioners resulted in a large degree of heterogeneity and few consistent relationships between barrier and strategy, meaning the relationship is rarely one-to-one (e.g., a single strategy is often is linked to multiple barriers; more than one strategy needed to address a single barrier). Moreover, when implementation outcomes are considered, researchers often find that to improve one outcome, more than one contextual barrier needs to be addressed, which might in turn require one or more strategies.

Frequently, the reporting of implementation research studies focuses on the strategy or strategies that were introduced for the research study, without due attention to other strategies already used in the system or additional supporting strategies that might be needed to implement the target strategy. The IRLM allows for the comprehensive specification of all introduced and present strategies, as well as their changes (adaptations, additions, discontinuations) during the project.

#### Mechanisms of action

Mechanisms of action are processes or events through which an implementation strategy operates to affect desired implementation outcomes [12]. The mechanism can be a change in a determinant, a proximal implementation outcome, an aspect of the implementation strategy itself, or a combination of these in a multiple-intervening-effect model. An example of a causal process might be using training and fidelity monitoring strategies to improve delivery agents’ knowledge and self-efficacy about the EBI in response to knowledge-related barriers in the service delivery system. This could result in raising their acceptability of the EBI, increase the likelihood of adoption, improve the fidelity of delivery, and lead to sustainment. Relatively, few implementation studies formally test mechanisms of action, but this area of investigation has received significant attention more recently as the necessity to understand how strategies operate grows in the field [33–35].

#### Outcomes

Implementation outcomes are the effects of deliberate and purposive actions to implement new treatments, practices, and services [21]. They can be indicators of implementation processes, or key intermediate outcomes in relation to service, or target clinical outcomes. Glasgow et al. [36–38] describe the interrelated nature of implementation outcomes as occurring in a logical, but not necessarily linear, sequence of adoption by a delivery agent, delivery of the innovation with fidelity, reach of the innovation to the intended population, and sustainment of the innovation over time. The combined impact of these nested outcomes, coupled with the size of the effect of the EBI, determines the population or public health impact of implementation [36]. Outcomes earlier in the sequence can be conceptualized as mediators and mechanisms of strategies on later implementation outcomes. Specifying which strategies are theoretically intended to affect which outcomes, through which mechanisms of action, is crucial for improving the rigor and reproducibility of implementation research and to testing theory.

### Using the Implementation Research Logic Model

#### Guiding principles

One of the critical insights from our preliminary work was that the use of the IRLM should be guided by a set of principles rather than governed by rules. These principles are intended to be flexible both to allow for adaptation to the various types of implementation studies and evolution of the IRLM over time and to address concerns in the field of implementation science regarding specification, rigor, reproducibility, and transparency of design and process [5]. Given this flexibility of use, the IRLM will invariably require accompanying text and other supporting documents. These are described in the section “Use of Supporting Text and Documents.”

#### Principle 1: Strive for *comprehensiveness*

Comprehensiveness increases transparency, can improve rigor, and allows for a better understanding of alternative explanations to the conclusions drawn, particularly in the presence of null findings for an experimental design. Thus, all relevant determinants, implementation strategies, and outcomes should be included in the IRLM.

#### Determinants

Concerning determinants, the valence should be noted as being either a barrier, a facilitator, neutral, or variable by study unit. This can be achieved by simply adding plus (+) or minus (–) signs for facilitators and barriers, respectively, or by using coding systems such as that developed by Damschroder et al. [39], which indicates the relative strength of the determinant on a scale: – 2 (*strong negative impact*), – 1 (*weak negative impact*), 0 (*neutral or mixed influence*), 1 (*weak positive impact*), and 2 (*strong positive impact*). The use of such a coding system could yield better specification compared to using study-specific adjectives or changing the name of the determinant (e.g., greater relative priority, addresses patient needs, good climate for implementation). It is critical to include all relevant determinants and not simply limit reporting to those that are hypothesized to be related to the strategies and outcomes, as there are complex interrelationships between determinants.

#### Implementation strategies

Implementation strategies should be reported in their entirety. When using the IRLM for planning a study, it is important to list all strategies in the system, including those already in use and those to be initiated for the purposes of the study, often in the experimental condition of the design. Second, strategies should be labeled to indicate whether they were (a) in place in the system prior to the study, (b) initiated prospectively for the purposes of the study (particularly for experimental study designs), (c) removed as a result of being ineffective or onerous, or (d) introduced during the study to address an emergent barrier or supplement other strategies because of low initial impact. This is relevant when using the IRLM for planning, as an ongoing tracking system, for retrospective application to a completed study, and in the final reporting of a study. There have been a number of processes proposed for tracking the use of and adaptations to implementation strategies over time [40, 41]. Each of these is more detailed than would be necessary for the IRLM, but the processes described provide a method for accurately tracking the temporal aspects of strategy use that fulfill the comprehensiveness principle.

#### Outcomes

Although most studies will indicate a primary implementation outcome, other outcomes are almost assuredly to be measured. Thus, they ought to be included in the IRLM. This guidance is given in large part due to the interdependence of implementation outcomes, such that adoption relates to delivery with fidelity, reach of the intervention, and potential for sustainment [36]. Similarly, the overall public health impact (defined as reach multiplied by the effect size of the intervention [38]) is inextricably tied to adoption, fidelity, acceptability, cost, etc. Although the study might justifiably focus on only one or two implementation outcomes, the others are nonetheless relevant and should be specified and reported. For example, it is important to capture potential unintended consequences and indicators of adverse effects that could result from the implementation of an EBI.

#### Principle 2: Indicate key *conceptual relationships*

Although the IRLM has a generalized theory (described earlier), there is a need to indicate the relationships between elements in a manner aligning with the specific theory of change for the study. Researchers ought to provide some form or notation to indicate these conceptual relationships using color-coding, superscripts, arrows, or a combination of the three. Such notations in the IRLM facilitate reference in the text to the study hypotheses, tests of effects, causal chain modeling, and other forms of elaboration (see “Supporting Text and Resources”). We prefer the use of superscripts to color or arrows in grant proposals and articles for practical purposes, as colors can be difficult to distinguish, and arrows can obscure text and contribute to visual convolution. When presenting the IRLM using presentation programs (e.g., PowerPoint, Keynote), colors and arrows can be helpful, and animations can make these connections dynamic and sequential without adding to visual complexity. This principle could also prove useful in synthesizing across similar studies to build the science of tailored implementation, where strategies are selected based on the presence of specific combinations of determinants. As previously indicated [29], there is much work to be done in this area given.

#### Principle 3: Specify critical *study design elements*

This critical element will vary by the study design (e.g., hybrid effectiveness-implementation trial, observational, what subsystems are assigned to the strategies). This principle includes not only researchers but service systems and communities, whose consent is necessary to carry out any implementation design [3, 42, 43].

#### Primary outcome(s)

Indicate the primary outcome(s) at each level of the study design (i.e., clinician, clinic, organization, county, state, nation). The levels should align with the specific aims of a grant application or the stated objective of a research report. In the case of a process evaluation or an observational study including the RE-AIM evaluation components [38] or the Proctor et al. [21] taxonomy of implementation outcomes, the primary outcome may be the product of the conceptual or theoretical model used when *a* priori outcomes are not clearly indicated. We also suggest including downstream health services and clinical outcomes even if they are not measured, as these are important for understanding the logic of the study and the ultimate health-related targets.

#### For quasi/experimental designs

When quasi/experimental designs [3, 4] are used, the independent variable(s) (i.e., the strategies that are introduced or manipulated or that otherwise differentiate study conditions) should be clearly labeled. This is important for internal validity and for differentiating conditions in multi-arm studies.

#### For comparative implementation trials

In the context of comparative implementation trials [3, 4], a study of two or more competing implementation strategies are introduced for the purposes of the study (i.e., the comparison is not implementation-as-usual), and there is a need to indicate the determinants, strategies, mechanisms, and potentially outcomes that differentiate the arms (see Additional File A2). As comparative implementation can involve multiple service delivery systems, the determinants, mechanisms, and outcomes might also differ, though there must be at least one comparable implementation outcome. In our preliminary work applying the IRLM to a large-scale comparative implementation trial, we found that we needed to use an IRLM for each arm of the trial as it was not possible to use a single IRLM because the strategies being tested occurred across two delivery systems and strategies were very different, by design. This is an example of the flexible use of the IRLM.

#### *For implementation optimization designs*

A number of designs are now available that aim to test processes of optimizing implementation. These include factorial, Sequential Multiple Assignment Randomized Trial (SMART) [44], adaptive [45], and roll-out implementation optimization designs [46]. These designs allow for (a) building time-varying adaptive implementation strategies based on the order in which components are presented [44], (b) evaluating the additive and combined effects of multiple strategies [44, 47], and (c) can incorporate data-driven iterative changes to improve implementation in successive units [45, 46]. The IRLM in Additional File A4 can be used for such designs.

#### Additional specification options

Users of the IRLM are allowed to specify any number of additional elements that may be important to their study. For example, one could notate those elements of the IRLM that have been or will be measured versus those that were based on the researcher’s prior studies or inferred from findings reported in the literature. Users can also indicate when implementation strategies differ by level or unit within the study. In large multisite studies, strategies might not be uniform across all units, particularly those strategies that already exist within the system. Similarly, there might be a need to increase the dose of certain strategies to address the relative strengths of different determinants within units.

### Using the IRLM for different purposes and stages of research

Commensurate with logic models more generally, the IRLM can be used for planning and organizing a project, carrying out a project (as a roadmap), reporting and presenting the findings of a completed project, and synthesizing the findings of multiple projects or of a specific area of implementation research, such as what is known about how learning collaboratives are effective within clinical care settings.

#### Planning

When the IRLM is used for planning, the process of populating each of the elements often begins with the known parameter(s) of the study. For example, if the problem is improving the adoption and reach of a specific EBI within a particular clinical setting, the implementation outcomes and context, as well as the EBI, are clearly known. The downstream clinical outcomes of the EBI are likely also known. Working from the two “bookends” of the IRLM, the researchers and community partners and/or organization stakeholders can begin to fill in the implementation strategies that are likely to be feasible and effective and then posit conceptually derived mechanisms of action. In another example, only the EBI and primary clinical outcomes were known. The IRLM was useful in considering different scenarios for what strategies might be needed and appropriate to test the implementation of the EBI in different service delivery contexts. The IRLM was a tool for the researchers and stakeholders to work through these multiple options.

#### Executing

When we used the IRLM to plan for the execution of funded implementation studies, the majority of the parameters were already proposed in the grant application. However, through completing the IRLM prior to the start of the study, we found that a number of important contextual factors had not been considered, additional implementation strategies were needed to complement the primary ones proposed in the grant, and mechanisms needed to be added and measured. At the time of award, mechanisms were not an expected component of implementation research projects as they will likely become in the future.

#### Reporting

For another project, the IRLM was applied retrospectively to report on the findings and overall logic of the study. Because nearly all elements of the IRLM were known, we approached completion of the model as a means of showing what happened during the study and to accurately report the hypothesized relationships that we observed. These relationships could be formally tested using causal pathway modeling [12] or other path analysis approaches with one or more intervening variables [48].

#### Synthesizing

In our preliminary work with the IRLM, we used it in each of the first three ways; the fourth (synthesizing) is ongoing within the National Cancer Institute’s Improving the Management of symPtoms during And Following Cancer Treatment (IMPACT) research consortium. The purpose is to draw conclusions for the implementation of an EBI in a particular context (or across contexts) that are shared and generalizable to provide a guide for future research and implementation.

#### Use of supporting text and documents

While the IRLM provides a good deal of information about a project in a single visual, researchers will need to convey additional details about an implementation research study through the use of supporting text, tables, and figures in grant applications, reports, and articles. Some elements that require elaboration are (a) preliminary data on the assessment and valence of implementation determinants; (b) operationalization/detailing of the implementation strategies being used or observed, using established reporting guidelines [9] and labeling conventions [32] from the literature; (c) hypothesized or tested causal pathways [12]; (d) process, service, and clinical outcome measures, including the psychometric properties, method, and timing of administration, respondents, etc.; (e) study procedures, including subject selection, assignment to (or observation of natural) study conditions, and assessment throughout the conduct of the study [4]; and (f) the implementation plan or process for following established implementation frameworks [49–51]. By utilizing superscripts, subscripts, and other notations within the IRLM, as previously suggested, it is easy to refer to (a) hypothesized causal paths in theoretical overviews and analytic plan sections, (b) planned measures for determinants and outcomes, and (c) specific implementation strategies in text, tables, and figures.

## Results

### Evidence of IRLM utility and acceptability

The IRLM was used as the foundation for a training in implementation research methods to a group of 65 planning projects awarded under the national Ending the HIV Epidemic initiative. One investigator (project director or co-investigator) and one implementation partner (i.e., a collaborator from a community service delivery system) from each project were invited to attend a 2-day in-person summit in Chicago, IL, in October 2019. One hundred thirty-two participants attended, representing 63 of the 65 projects. A survey, which included demographics and questions pertaining to the Ending the HIV Epidemic, was sent to potential attendees prior to the summit, to which 129 individuals—including all 65 project directors, 13 co-investigators, and 51 implementation partners (62% Female)—responded. Those who indicated an investigator role (*n* = 78) received additional questions about prior implementation research training (e.g., formal coursework, workshop, self-taught) and related experiences (e.g., involvement in a funded implementation project, program implementation, program evaluation, quality improvement) and the stage of their project (i.e., exploration, preparation, implementation, sustainment [50]).

Approximately 6 weeks after the summit, 89 attendees (69%) completed a post-training survey comprising more than 40 questions about their overall experience. Though the invitation to complete the survey made no mention of the IRLM, it included 10 items related to the IRLM and one more generally about the logic of implementation research, each rated on a 4-point scale (1 = *not at all*, 2 = *a little*, 3 = *moderately*, 4 = *very much*; see Table 1). Forty-two investigators (65% of projects) and 24 implementation partners indicated attending the training and began and completed the survey (68.2% female). Of the 66 respondents who attended the training, 100% completed all 11 IRLM items, suggesting little potential response bias.

**Table 1:** Survey results

Question			
To what extent was the Implementation Research Logic Model (IRLM) helpful in…	Mean	*SD*	% responding either “Moderately” or “Very”
improving the rigor and reproducibility	3.05	.885	77.7%
serving as a “roadmap” for how the project is to be carried out over time	3.08	.950	74.0%
clearly reporting and specifying how the project is to be conducted	2.94	.909	67.8%
understanding the connections between determinants, strategies, mechanisms, and outcomes	2.92	.957	66.3%
identifying gaps in the implementation research logic of their project	2.86	1.021	64.2%
deepening their knowledge of implementation science methods	2.83	.959	62.9%
planning the project	2.82	1.088	61.3%
developing consensus and understanding of the project among diverse stakeholders involved	2.75	1.090	58.8%
identifying gaps in new research questions or analyses	2.54	1.032	51.3%
To what extent…			
… were the worksheets provided during the summit helpful in completing the IRLM	3.02	.886	74.1%
… has your knowledge on the logic of implementation research increased after the two-day training	3.18	.827	77.6%

Table 1 provides the means, standard deviations, and percent of respondents endorsing either “moderately” or “very” response options. Results were promising for the utility of the IRLM on the majority of the dimensions assessed. More than 50% of respondents indicated that the IRLM was “moderately” or “very” helpful on all questions. Overall, 77.6% (*M* = 3.18, *SD* = .827) of respondents indicated that their knowledge on the logic of implementation research had increased either moderately or very much after the 2-day training. At the time of the survey, when respondents were about 2.5 months into their 1-year planning projects, 44.6% indicated that they had already been able to complete a full draft of the IRLM.

Additional analyses using a one-way analysis of variance indicated no statistically significant differences in responses to the IRLM questions between investigators and implementation partners. However, three items approached significance: planning the project (*F* = 2.460, *p* = .055), clearly reporting and specifying how the project is to be conducted (*F* = 2.327, *p* = .066), and knowledge on the logic of implementation research (*F* = 2.107, *p* = .091). In each case, scores were higher for the investigators compared to the implementation partners, suggesting that perhaps the knowledge gap in implementation research lay more in the academic realm than among community partners, who may not have a focus on research but whose day-to-day roles include the implementation of EBPs in the real world. Lastly, analyses using ordinal logistic regression did not yield any significant relationship between responses to the IRLM survey items and prior training (*n* = 42 investigators who attended the training and completed the post-training survey), prior related research experience (*n* = 42), and project stage of implementation (*n* = 66). This suggests that the IRLM is a useful tool for both investigators and implementers with varying levels of prior exposure to implementation research concepts and across all stages of implementation research. As a result of this training, the IRLM is now a required element in the FY2020 Ending the HIV Epidemic Centers for AIDS Research/AIDS Research Centers Supplement Announcement released March 2020 [15].

### Resources for using the IRLM

As the use of the IRLM for different study designs and purposes continues to expand and evolve, we envision supporting researchers and other program implementers in applying the IRLM to their own contexts. Our team at Northwestern University hosts web resources on the IRLM that includes completed examples and tools to assist users in completing their model, including templates in various formats (Figs. 1 and 2, Additional Files A1, A2, A3 and A4 and others) a Quick Reference Guide (Additional File A8) and a series of worksheets that provide guidance on populating the IRLM (Additional File A9). These will be available at https://cepim.northwestern.edu/implementationresearchlogicmodel/.

## Discussion

The IRLM provides a compact visual depiction of an implementation project and is a useful tool for academic–practice collaboration and partnership development. Used in conjunction with supporting text, tables, and figures to detail each of the primary elements, the IRLM has the potential to improve a number of aspects of implementation research as identified in the results of the post-training survey. The usability of the IRLM is high for seasoned and novice implementation researchers alike, as evidenced by our survey results and preliminary work. Its use in the planning, executing, reporting, and synthesizing of implementation research could increase the rigor and transparency of complex studies that ultimately could improve reproducibility—a challenge in the field—by offering a common structure to increase consistency and a method for more clearly specifying links and pathways to test theories.

Implementation occurs across the gamut of contexts and settings. The IRLM can be used when large organizational change is being considered, such as a new strategic plan with multifaceted strategies and outcomes. Within a narrower scope of a single EBI in a specific setting, the larger organizational context still ought to be included as inner setting determinants (i.e., the impact of the organizational initiative on the specific EBI implementation project) and as implementation strategies (i.e., the specific actions being done to make the organizational change a reality that could be leveraged to implement the EBI or could affect the success of implementation). The IRLM has been used by our team to plan for large systemic changes and to initiate capacity building strategies to address readiness to change (structures, processes, individuals) through strategic planning and leadership engagement at multiple levels in the organization. This aspect of the IRLM continues to evolve.

Among the drawbacks of the IRLM is that it might be viewed as a somewhat simplified format. This represents the challenges of balancing depth and detail with parsimony, ease of comprehension, and ease of use. The structure of the IRLM may inhibit creative thinking if applied too rigidly, which is among the reasons we provide numerous examples of different ways to tailor the model to the specific needs of different project designs and parameters. Relatedly, we encourage users to iterate on the design of the IRLM to increase its utility.

## Conclusions

The promise of implementation science lies in the ability to conduct rigorous and reproducible research, to clearly understand the findings, and to synthesize findings from which generalizable conclusions can be drawn and actionable recommendations for practice change emerge. As scientists and implementers have worked to better define the core methods of the field, the need for theory-driven, testable integration of the foundational elements involved in impactful implementation research has become more apparent. The IRLM is a tool that can aid the field in addressing this need and moving toward the ultimate promise of implementation research to improve the provision and quality of healthcare services for all people.

## Supplementary information

**Additional file 1.** IRLM Fillable PDF form

**Additional file 2.** IRLM for Comparative Implementation

**Additional file 3.** IRLM for Implementation of an Intervention Across or Linking Two Contexts

**Additional file 4.** IRLM for an Implementation Optimization Study

**Additional file 5.** IRLM example 1: Faith in Action: Clergy and Community Health Center Communication Strategies for Ending the Epidemic in Mississippi and Arkansas

**Additional file 6.** IRLM example 2: Hybrid Type II Effectiveness–Implementation Evaluation of a City-Wide HIV System Navigation Intervention in Chicago, IL

**Additional file 7.** IRLM example 3: Implementation, spread, and sustainment of Physical Therapy for Mild Parkinson’s Disease through a Regional System of Care

**Additional file 8.** IRLM Quick Reference Guide

**Additional file 9.** IRLM Worksheets

## Data Availability

Not applicable.

## Abbreviations

CFIR: Consolidated Framework for Implementation Research
EBI: Evidence-based intervention
ERIC: Expert Recommendations for Implementing Change
IRLM: Implementation Research Logic Model
