# Supplementary figures and images for: The Implementation Research Logic Model: a method for planning, executing, reporting, and synthesizing implementation projects
Source: Implement Sci. 2020 Sep 25;15:84. doi: 10.1186/s13012-020-01041-8 (PMC7523057; doi:10.1186/s13012-020-01041-8)

# Implementation Research Logic Model (IRLM)

Project  
Title:

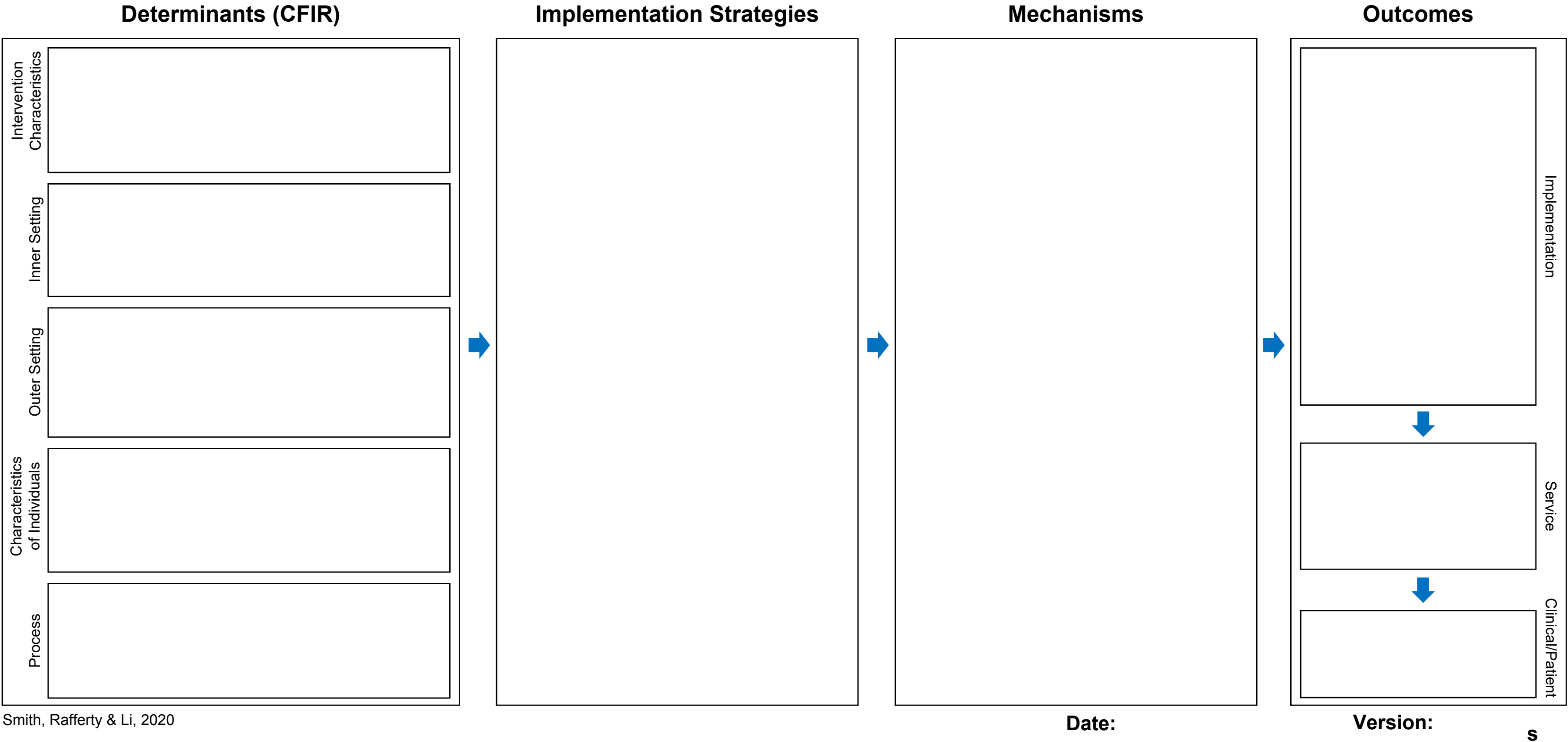

Supplement: Supplementary file 1 — Additional file 1. IRLM Fillable PDF form [file 13012_2020_1041_MOESM1_ESM.pdf]

# IR Logic Model for Implementation Optimization Trial (4 clusters; 1 setting)

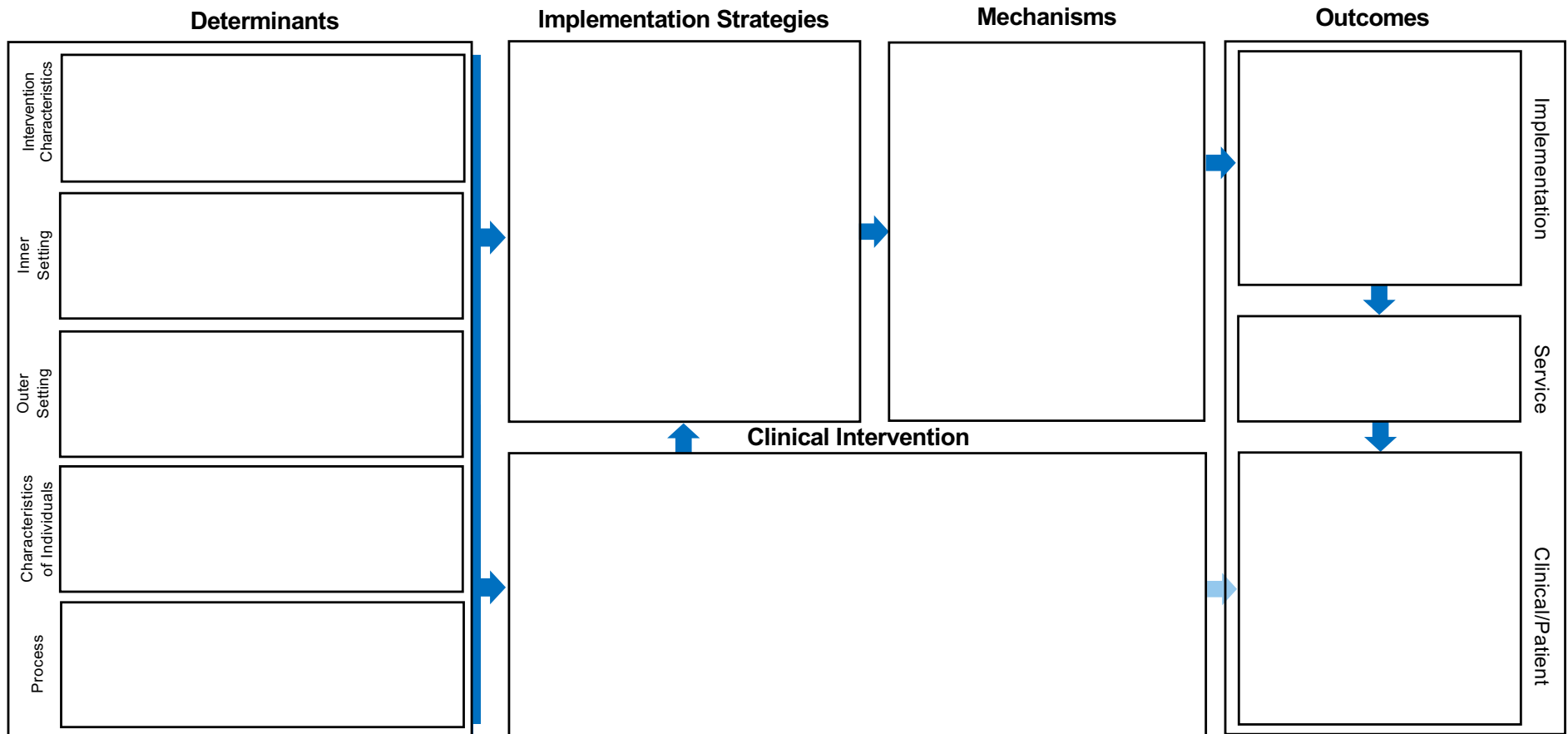

Supplement: Supplementary file 2 — Additional file 2. IRLM for Comparative Implementation [file 13012_2020_1041_MOESM2_ESM.pdf]

# IRLM for Multi-Context Implementation of Single Intervention

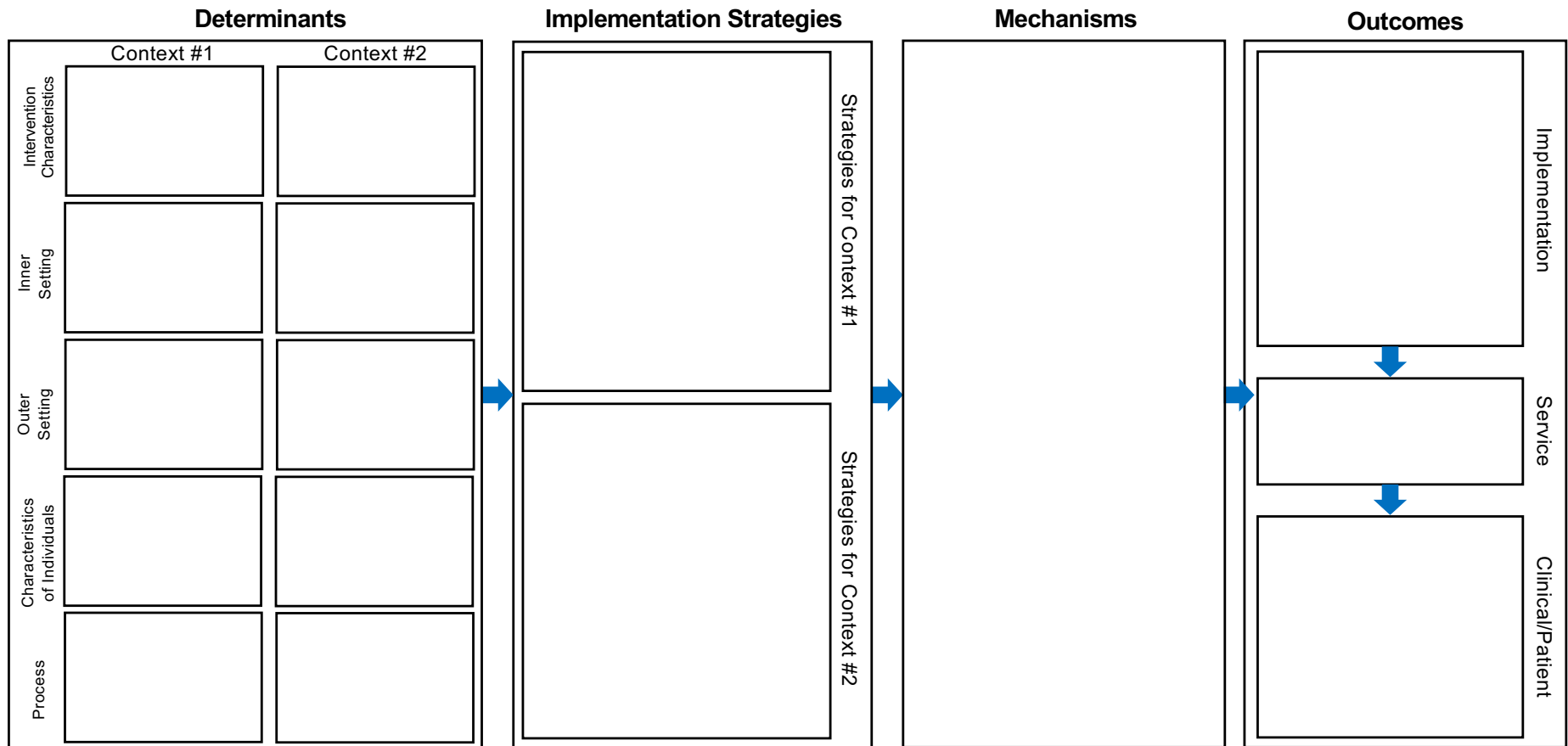

Supplement: Supplementary file 3 — Additional file 3. IRLM for Implementation of an Intervention Across or Linking Two Contexts [file 13012_2020_1041_MOESM3_ESM.pdf]

# IRLM for Implementation Optimization Trial

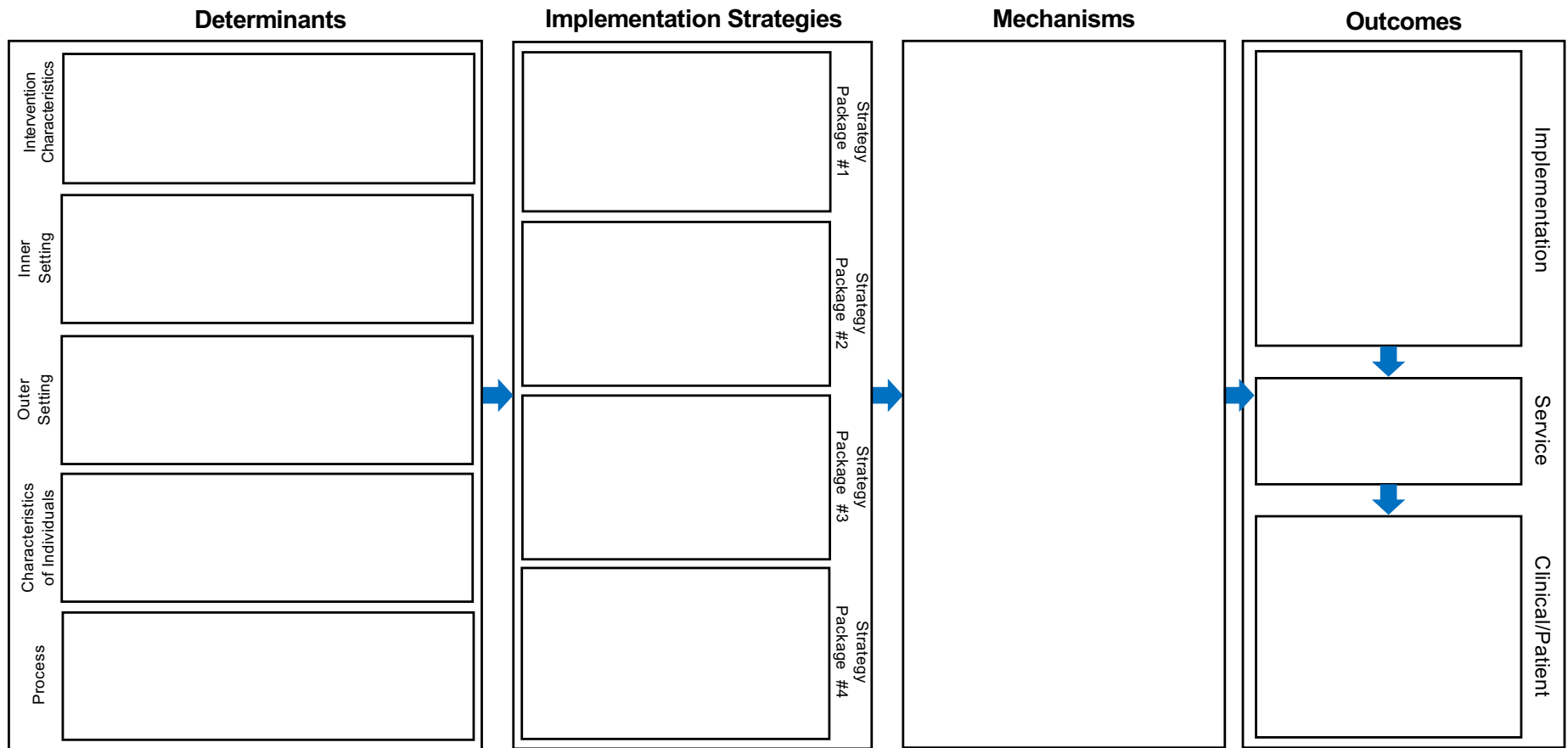

Supplement: Supplementary file 4 — Additional file 4. IRLM for an Implementation Optimization Study [file 13012_2020_1041_MOESM4_ESM.pdf]

# IRLM Quick Reference Guide

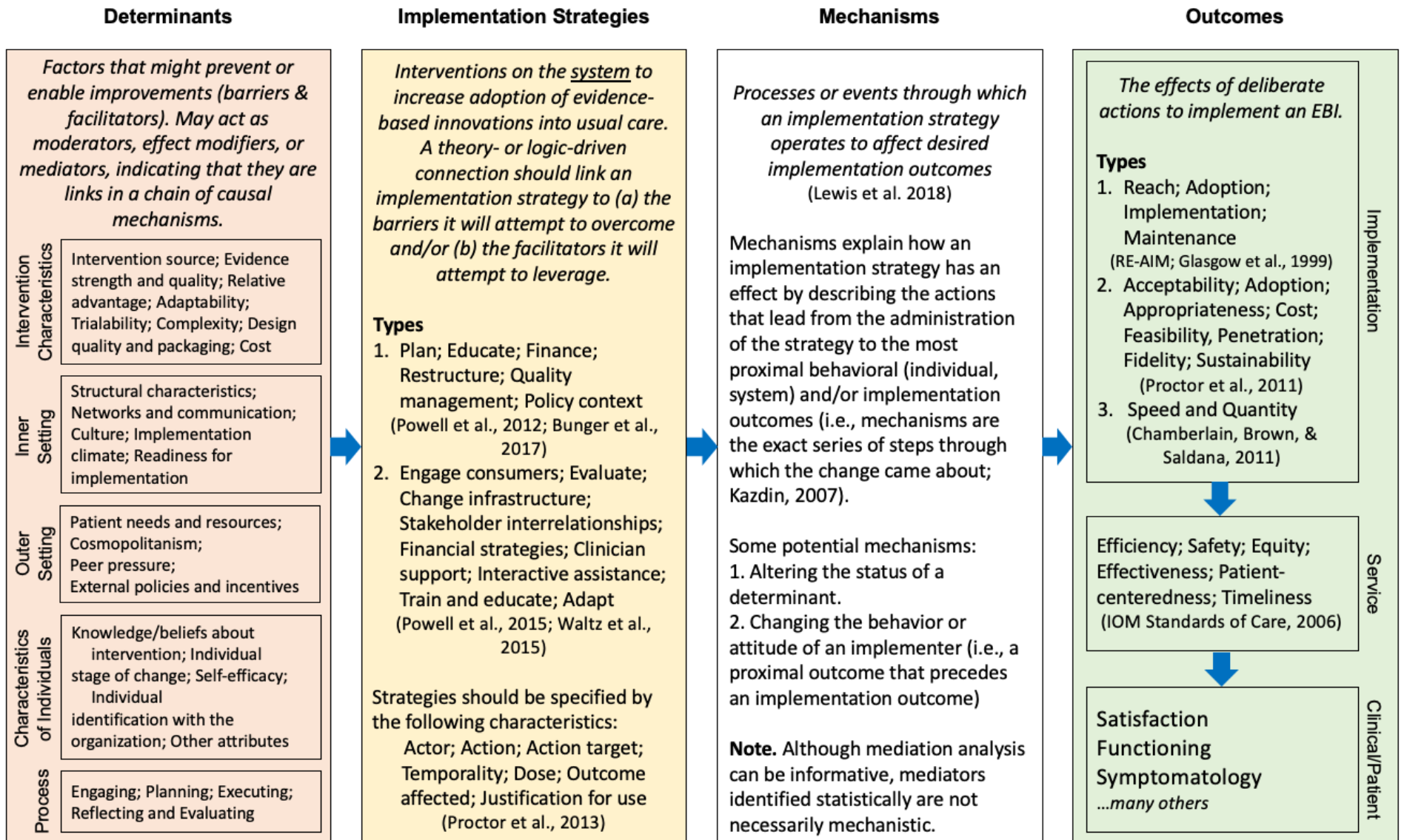

Supplement: Supplementary file 8 — Additional file 8. IRLM Quick Reference Guide [file 13012_2020_1041_MOESM8_ESM.pdf]
