# Supplementary material for: The Implementation Research Logic Model: a method for planning, executing, reporting, and synthesizing implementation projects
Source: Implement Sci. 2020 Sep 25;15:84. doi: 10.1186/s13012-020-01041-8 (PMC7523057; doi:10.1186/s13012-020-01041-8)
Supplement: Supplementary file 5 — Additional file 5. IRLM example 1: Faith in Action: Clergy and Community Health Center Communication Strategies for Ending the Epidemic in Mississippi and Arkansas [file 13012_2020_1041_MOESM5_ESM.pdf]

Additional File A5

Title: Faith in Action: Clergy and Community Health Center Communication Strategies for Ending the Epidemic in Mississippi and Arkansas
Principal Investigator: Nunn, Amy (Brown University)
FOA: One-Year FY2020 EHE CFAR/ARC Supplement Announcement
Status: Awarded

Project Description: Nearly half of new HIV infections occur in the Southeastern United States and most new infections are among African Americans. Mississippi and Arkansas are two states named in the Ending the HIV Epidemic (EtHE) that face significant rural HIV burdens. Little HIV research has been conducted in rural areas in these states, and many rural communities with significant HIV burden are medically underserved.[9] There is tremendous unmet need for routine HIV screening, HIV prevention and care services in these southern states. Partnering with faith-based organizations is also an important component of the EtHE "all of society" approach to ending the epidemic. There is public health opportunity to leverage the power of African American faith-based organizations to reduce racial disparities in the HIV and pre-exposure prophylaxis (PrEP) care continua. There is also opportunity to engage clergy in partnerships with community health centers in rural areas. Community health centers will be key EtHE partners for scaling HIV screening, PrEP and HIV care in the rural South. We propose the following specific aims:

Aim 1. Develop social marketing materials featuring African American clergy that promote HIV screening, PrEP and HIV care at community health centers in geographic hotspots of HIV infection in Arkansas and Mississippi. These social marketing materials may include videos, social media, pamphlets and palm cards used in community health center lobbies and by community health center personnel to normalize and promote uptake of routine HIV screening and HIV care.

Aim 2. Pilot test and assess the implementation and acceptability of the social marketing campaign at two community health centers in Arkansas and Mississippi. Findings will be used to develop an implementation strategy for increasing uptake and acceptability of routine HIV screening and HIV care at community health centers. Findings will also inform best practices for building partnerships between community health centers and faith institutions.

Notes about IRLM use in this project: The IRLM was useful in specifying the <u>primary</u> implementation strategies being proposed and linking these to preliminary data on the context (determinants with valence coding based on data and community partner report) and operationalizing the implementation outcomes for this one-year supplement award. Additionally, measurable mechanisms were hypothesized that otherwise would not have been included in the proposal. Additionally, a justification was included within the IRLM for each of the primary strategies. A comprehensive IRLM (complete implementation strategies and mechanisms) will be developed as the project progresses.

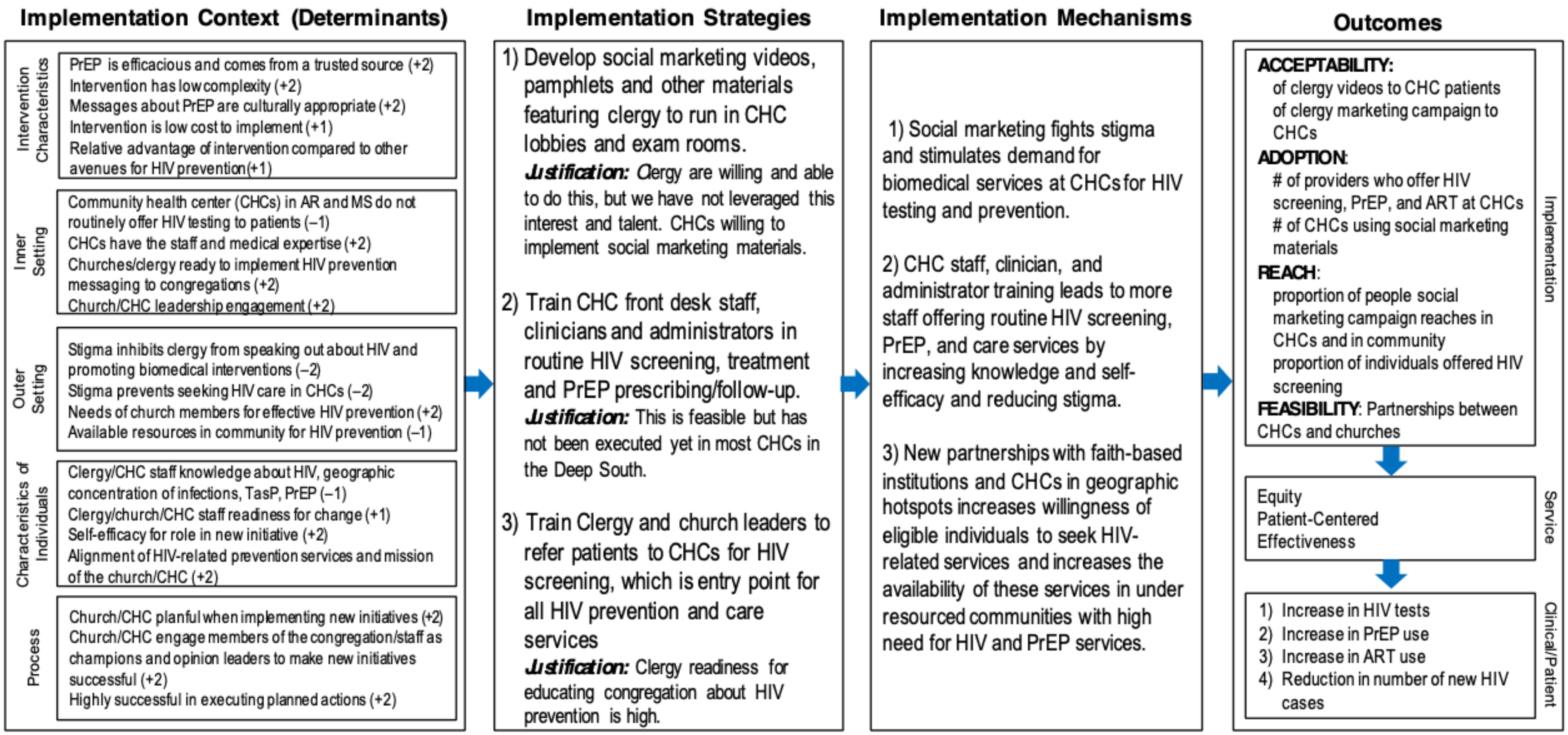

Notes. ART = antiretroviral treatment. PreP = Pre-exposure prophylaxis. TasP = treatment as prevention. CHC = community health center.
