# Supplementary material for: The Implementation Research Logic Model: a method for planning, executing, reporting, and synthesizing implementation projects
Source: Implement Sci. 2020 Sep 25;15:84. doi: 10.1186/s13012-020-01041-8 (PMC7523057; doi:10.1186/s13012-020-01041-8)
Supplement: Supplementary file 6 — Additional file 6. IRLM example 2: Hybrid Type II Effectiveness–Implementation Evaluation of a City-Wide HIV System Navigation Intervention in Chicago, IL [file 13012_2020_1041_MOESM6_ESM.pdf]

## Additional File A6

**Title:** Hybrid Type II Effectiveness–Implementation Evaluation of a City-Wide HIV System Navigation Intervention in Chicago, IL
**Principal Investigator:** Li, Dennis (Northwestern University)
**FOA:** One-Year FY2020 EHE CFAR/ARC Supplement Announcement
**Status:** Not Awarded

**Project Description:** HIV system navigation (HSN) is an evidence-based, CDC-endorsed service delivery model for improving linkage to and successful use of HIV healthcare, prevention, and ancillary services (e.g., housing, substance abuse treatment) among people living with and vulnerable to HIV. Notably, it effectively reduces disparities in access among clients traditionally considered "hard to reach" due to trauma, comorbidities, or other barriers to care. Guided by this model, the Chicago Department of Public Health funded the development of a coordinated HSN program for the Chicago metropolitan area. The new HIV Resource Coordination Hub is unique and innovative among HSN programs, however, because of its centralized role: Once fully implemented, the Hub will integrate information and referrals across organizations, target populations, and service sectors to become "the only door a person needs" to access all HIV-related services within the city. Expanding HNS to encompass and bridge multiple providers and systems in a large metro area has the potential to increase optimization of HIV service utilization and resources and to reach communities previously missed, but new implementation challenges may arise from the increased complexity, additional stakeholders (i.e., providers), and other factors. Formative research to understand contextual determinants of and strategies for implementing the Hub is critical to ensure that this novel approach is able to deliver on its potential. Our specific aims are:

- **Aim 1.** Evaluate the effectiveness of the Hub on service- and patient-level outcomes.
- **Aim 2.** Optimize strategies to improve implementation and reach of the Hub.
- **Aim 3.** Explore contextual factors and strategies that may influence early/late adoption of the Hub among HIV service providers.

**Notes about IRLM use in this project:** The IRLM was useful for comprehensively documenting all of the potential determinants of implementation among the three primary stakeholder groups and linking them to corresponding levels of outcomes to be assessed in the study. In collaboration with the implementation partner, we were able to organize and specify primary and secondary implementation strategies as well as differentiate between existing and potential strategies. The alternative format also allowed us to better describe components of the intervention in the context of the other implementation research components.

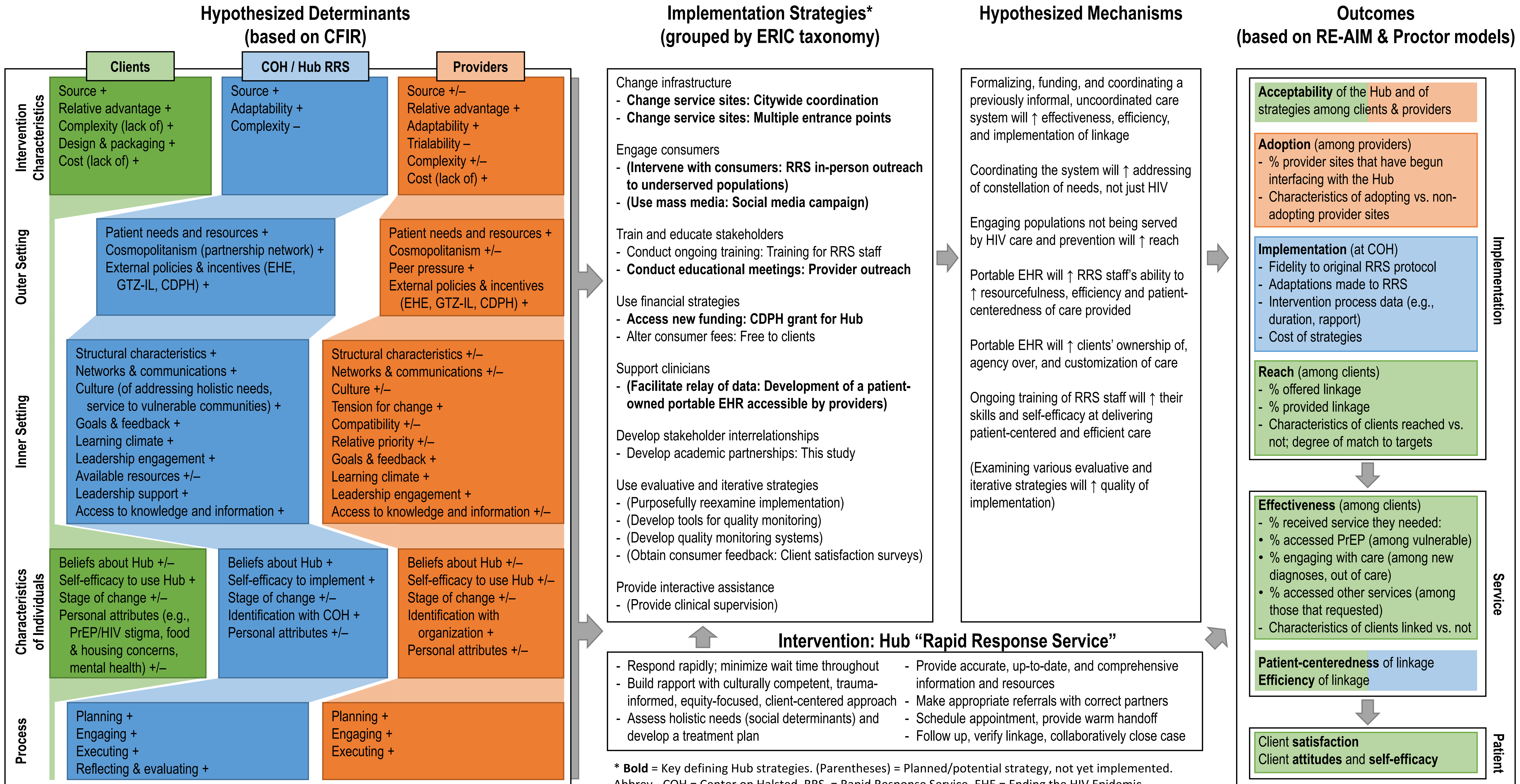


* **Bold** = Key defining Hub strategies. (Parentheses) = Planned/potential strategy, not yet implemented.
Abbrev. COH = Center on Halsted. RRS = Rapid Response Service. EHE = Ending the HIV Epidemic.
GTZ-IL = Getting to Zero Illinois. CDPH = Chicago Department of Public Health. +/– = facilitator/barrier.
