# Supplementary material for: The Implementation Research Logic Model: a method for planning, executing, reporting, and synthesizing implementation projects
Source: Implement Sci. 2020 Sep 25;15:84. doi: 10.1186/s13012-020-01041-8 (PMC7523057; doi:10.1186/s13012-020-01041-8)
Supplement: Supplementary file 7 — Additional file 7. IRLM example 3: Implementation, spread, and sustainment of Physical Therapy for Mild Parkinson’s Disease through a Regional System of Care [file 13012_2020_1041_MOESM7_ESM.pdf]

Additional File A7

Title: Implementation, spread, and sustainment of Physical Therapy for Mild Parkinson's Disease through a Regional System of Care

Principal Investigator: Rafferty, Miriam (Shirley Ryan AbilityLab, Northwestern University)
FOA: Parkinson's Foundation Care Improvement Initiative (2016-2017); Academy of Neurologic Physical Therapy Knowledge Translation Summit Grant (2018-2020)
Status: Awarded

Project Description: Parkinson's disease is a chronic condition that causes progressive mobility impairments associated with decreased quality of life and increased mortality. Clinical practice guidelines recommend outpatient physical therapy early in the disease process in order instruct in an individually tailored physical activity and exercise program[1]. Our central goal and purpose for this project was to implement a sustainable proactive physical therapy program throughout a regional system of care for people with early stage Parkinson's disease with the goal to help them increase and maintain optimal exercise and physical activity levels. We call this program Proactive Physical Therapy. In 2016, we had implemented a small-scale pilot of Proactive Physical Therapy with two clinicians at one site[2]. Then in 2018-2019, we implemented a sustainability project at the initial, urban academic site as well as two smaller community clinics. Our program aims at all three sites included:

> **Aim 1 (Process Outcome):** To improve access (reach, adoption) to individualized exercise prescription and monitoring in people with early Parkinson's disease using a long-term Proactive Physical Therapy approach.
>
> **Aim 2 (Clinical Outcome):** To improve participation in physical activity, including moderate to vigorous intensity exercise, in people with early Parkinson's disease.

Notes about IRLM use in this project: The IRLM was applied after completing the sustainment study to aid in describing how determinants, implementation strategies, and mechanisms were related. The initial project was structured using The Knowledge-to-Action Cycle process framework,[3] the CFIR determinants framework,[2] and the RE-AIM evaluation framework.[4] The use of these frameworks enabled the program to address our context-specific needs as well as further understand how these needs vary between different clinics. Addition of the IRLM after local study completion will aid in communication during grant applications for future multisite trials. In particular, the example IRLM with a clinical intervention specified will help communicate the differences and relationships between the clinical evidence-based intervention and some of the key implementation strategies or implementation interventions that may studied.

References:

1. Keus S MM, Graziano M, et al. . European Physiotherapy Guideline for Parkinson's disease. *KNGF/ParkinsonNet.* 2014.
2. Rafferty MR, MacDonald J, Byskosh A, et al. Using Implementation Frameworks to Provide Proactive Physical Therapy for People With Parkinson Disease: Case Report. *Phys Ther.* 2019;99(12):1644-1655.
3. Graham ID, Logan J, Harrison MB, et al. Lost in knowledge translation: time for a map? *J Contin Educ Health Prof.* 2006;26(1):13-24.
4. Glasgow, R. E., Harden, S. M., Gaglio, B., Rabin, B., Smith, M. L., Porter, G. C., ... & Estabrooks, P. A. (2019). RE-AIM planning and evaluation framework: adapting to new science and practice with a 20-year review. *Frontiers in public health*, *7*, 64.

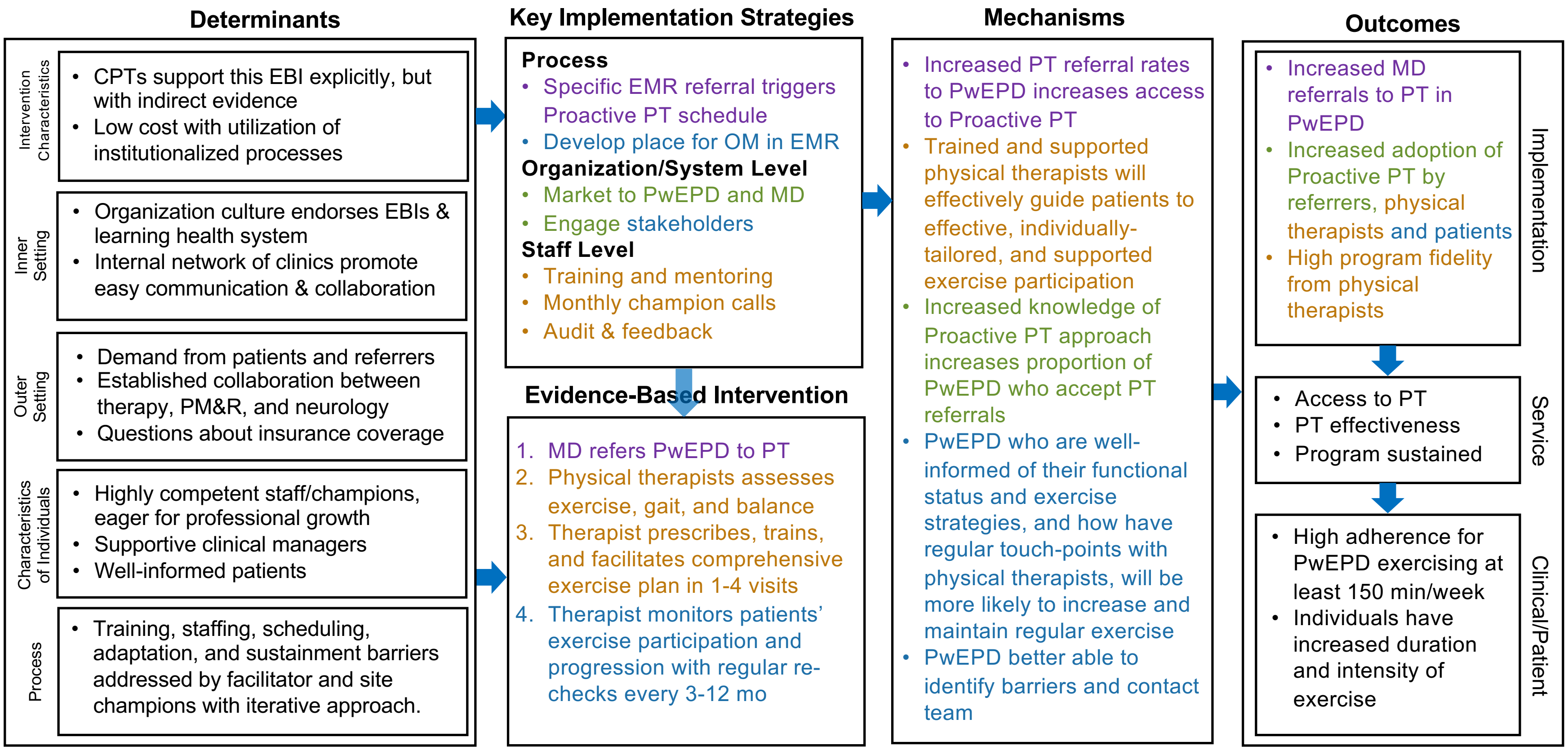

Abbreviations: CPG=clinical practice guidelines; EBI=evidenced based intervention; EMR= electronic medical record; MD= medical doctor; OM=outcome measures; PM&R=physical medicine and rehabilitation; PwEPD=people with early Parkinson's Disease; PT=physical therapy

Reference: Rafferty MR, MacDonald J, Byskosh A, Sloan L, Toledo S, Marciniak C, et al. Using Implementation Frameworks to Provide Proactive Physical Therapy for People With Parkinson Disease: Case Report. Phys Ther. 2019;99(12):1644-55.

Notes. Colors indicate conceptual and theoretical links in a causal chain.
