# Supplementary material for: The Implementation Research Logic Model: a method for planning, executing, reporting, and synthesizing implementation projects
Source: Implement Sci. 2020 Sep 25;15:84. doi: 10.1186/s13012-020-01041-8 (PMC7523057; doi:10.1186/s13012-020-01041-8)
Supplement: Supplementary file 9 — Additional file 9. IRLM Worksheets [file 13012_2020_1041_MOESM9_ESM.pdf]

IRLM — Determinants of Implementation Worksheet

Smith, Li, & Rafferty, 2020

Determinants of implementation are constructs that have been associated with effective implementation. Often, researchers think of determinants as implementation barriers and facilitators, but they can also be mediators, moderators, predictors, and/or outcomes. One of the most comprehensive lists of determinants comes from the Consolidated Framework for Implementation Research (CFIR; Damschroder et al., 2009).

1. From the list of CFIR constructs below, place a checkmark (√) next to ones that may be germane to your project. It is important to capture all factors that may affect the implementation of your intervention.

2. Circle any determinants that your project may aim to change/alter.

3. For each determinant, operationalize it for your project and add it to your **IRLM**.

√	Determinant	Definition
	**Intervention Characteristics**	
	Intervention source	Perception of key stakeholders about whether the intervention is externally or internally developed.
	Evidence strength and quality	Stakeholders' perceptions of the quality and validity of evidence supporting the belief that the intervention will have desired outcomes.
	Relative advantage	Stakeholders' perception of the advantage of implementing the intervention versus an alternative solution.
	Adaptability	The degree to which an intervention can be adapted, tailored, refined, or reinvented to meet local needs.
	Trialability	The ability to test the intervention on a small scale in the organization, and to be able to reverse course (undo implementation) if warranted.
	Complexity	Perceived difficulty of the intervention, reflected by duration, scope, radicalness, disruptiveness, centrality, and intricacy and number of steps required to implement.
	Design quality and packaging	Perceived excellence in how the intervention is bundled, presented, and assembled.
	Cost	Costs of the intervention and costs associated with implementing the intervention including investment, supply, and opportunity costs.
	**Outer Setting**	
	Patient needs and resources	The extent to which patient needs, as well as barriers and facilitators to meet those needs, are accurately known and prioritized by the organization.
	Cosmopolitanism	The degree to which an organization is networked with other external organizations.
	Peer pressure	Mimetic or competitive pressure to implement an intervention; typically because most or other key peer or competing organizations have already implemented or are in a bid for a competitive edge.
	External policies and incentives	A broad construct that includes external strategies to spread interventions, including policy and regulations (governmental or other central entity), external mandates, recommendations and guidelines, pay-for-performance, collaboratives, and public or benchmark reporting.

	**Inner Setting**	
	Structural characteristics	The social architecture, age, maturity, and size of an organization.
	Networks and communication	The nature and quality of webs of social networks and the nature and quality of formal and informal communications within an organization.
	Culture	Norms, values, and basic assumptions of a given organization.
	Implementation climate<br>- *Tension for change*<br>- *Compatibility*<br>- *Relative priority*<br>- *Incentives & rewards*<br>- *Goals and feedback*<br>- *Learning climate*	The absorptive capacity for change, shared receptivity of involved individuals to an intervention, and the extent to which use of that intervention will be rewarded, supported, and expected within their organization.
	Readiness for implementation<br>- *Leadership engagement*<br>- *Available resources*<br>- *Access to knowledge*	Tangible and immediate indicators of organizational commitment to its decision to implement an intervention.
	**Characteristics of Individuals**	
	Knowledge/beliefs about intervention	Individuals' attitudes toward and value placed on the intervention as well as familiarity with facts, truths, and principles related to the intervention.
	Individual stage of change	Characterization of the phase an individual is in, as he or she progresses toward skilled, enthusiastic, and sustained use of the intervention.
	Self-efficacy	Individual belief in their own capabilities to execute courses of action to achieve implementation goals.
	Individual identification with the organization	A broad construct related to how individuals perceive the organization, and their relationship and degree of commitment with that organization.
	Other attributes	A broad construct to include other personal traits such as tolerance of ambiguity, intellectual ability, motivation, values, competence, capacity, and learning style.
	**Process**	
	Engaging<br>- *Opinion leaders*<br>- *Formal internal implementation leaders*<br>- *Champions*<br>- *External change agents*	Attracting and involving appropriate individuals in the implementation and use of the intervention through a combined strategy of social marketing, education, role modeling, training, and other similar activities.
	Planning	The degree to which a scheme or method of behavior and tasks for implementing an intervention are developed in advance, and the quality of those schemes or methods.
	Executing	Carrying out or accomplishing the implementation according to plan.
	Reflecting and evaluating	Quantitative and qualitative feedback about the progress and quality of implementation accompanied with regular personal and team debriefing about progress and experience.

IRLM — Implementation Outcomes Worksheet

Smith, Li, & Rafferty, 2020

Implementation outcomes are "the effects of deliberate and purposive actions to implement new treatments, practices, and services" (Proctor et al., 2011). They serve as (1) indicators of implementation success, (2) proximal indicators of implementation processes, and (3) *intermediate outcomes in relation to service and clinical/patient outcomes*:

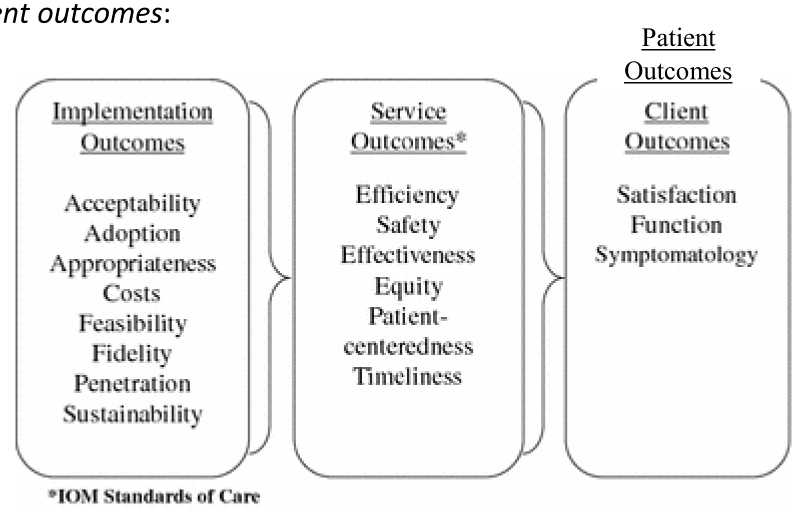

Adapted from Proctor et al. 2011

Unlike clinical/patient outcomes, implementation outcomes are often at the level of the system, setting, or service provider and typically not at the level of the patient/client. Some outcomes may be measured by researchers, whereas other may be measured through administrative records.

To identify implementation outcomes for your project, it is helpful to work backward from the most downstream/ distal/long-term to more upstream/proximal/short-term outcomes.

1. For the evidence-based intervention that is the focus of your project, what are the clinical/patient outcomes you are interested in? These may include clinical indicators, patient behaviors, patient-reported outcomes, etc. Add these to your **IRLM**.

2. From the list of service outcomes below, place a checkmark (√) next to ones that may be relevant to your project. Add these to your **IRLM**.

√	Service outcome	Definition
	Efficiency	Avoiding waste, including waste of equipment, supplies, ideas, and energy.
	Safety	Avoiding harm to patients from the care that is intended to help them.
	Effectiveness	Providing services based on scientific knowledge to all who could benefit and refraining from providing services to those not likely to benefit (avoiding underuse and misuse, respectively).
	Equity	Providing care that does not vary in quality because of personal characteristics such as gender, ethnicity, geographic location, and socioeconomic status.
	Patient-centeredness	Providing care that is respectful of and responsive to individual patient preferences, needs, and values and ensuring that patient values guide all clinical decisions.
	Timeliness	Reducing waits and sometimes harmful delays for both those who receive and those who give care.

3. From the list of implementation outcomes below, place a checkmark (√) next to ones that may be germane to your project. For each outcome, operationalize it for your project and add it to your **IRLM.**

√	Implementation outcome	Definition
	**RE-AIM Framework (www.re-aim.org; Glasgow, Vogt, & Boles, 1999)**	
	Reach	The absolute number, proportion, and representativeness of individuals who are willing to participate in a given initiative, intervention, or program.
	(Effectiveness)	(See service outcomes.) The impact of an intervention on important outcomes, including potential negative effects, quality of life, and economic outcomes.
	Adoption	The absolute number, proportion, and representativeness of settings and intervention agents (people who deliver the program) who are willing to initiate a program.
	Implementation	At the setting level, implementation refers to the intervention agents' fidelity to the various elements of an intervention's protocol, including consistency of delivery as intended and the time and cost of the intervention. At the individual level, implementation refers to clients' use of the intervention strategies.
	Maintenance	The extent to which a program or policy becomes institutionalized or part of the routine organizational practices and policies. Within the RE-AIM framework, maintenance also applies at the individual level. At the individual level, maintenance has been defined as the long-term effects of a program on outcomes after 6 or more months after the most recent intervention contact.
	**Proctor et al., 2011**	
	Acceptability	The perception among implementation stakeholders that a given treatment, service, practice, or innovation is agreeable, palatable, or satisfactory.
	Adoption	The intention, initial decision, or action to try or employ an innovation or evidence-based practice.
	Appropriateness	The perceived fit, relevance, or compatibility of the innovation or evidence based practice for a given practice setting, provider, or consumer; and/or perceived fit of the innovation to address a particular issue or problem.
	Cost	The cost impact of an implementation effort.
	Feasibility	The extent to which a new treatment, or an innovation, can be successfully used or carried out within a given agency or setting.
	Fidelity	The degree to which an intervention was implemented as it was prescribed in the original protocol or as it was intended by the program developers.
	Penetration/Uptake	The integration of a practice within a service setting and its subsystems.
	Sustainability	The extent to which a newly implemented treatment is maintained or institutionalized within a service setting's ongoing, stable operation.

IRLM — Implementation Strategies Worksheet

Smith, Li, & Rafferty, 2020

In implementation research, the word "intervention" can refer to two things:

- An evidence-based intervention→ the program, policy, practice, pill, etc., that affects patient outcomes.
- An implementation intervention→ manipulations to the *system* that help implement the EBI.

To avoid inevitable confusion, we typically refer to the latter as "strategies."

When implementing an EBI, multiple discrete strategies are typically used. Several taxonomies/lists of strategies exist in the literature, including by Bunger et al. (2017) and by Powell et al. (2015).

1. From either taxonomy below, place a checkmark (√) next to strategy categories that you may be considering for your project.
    a. For help selecting strategies based on your determinants of implementation, you may use the CFIR-ERIC Matching Tool found at https://cfirguide.org/choosing-strategies/.
2. For each strategy category, identify discrete strategies and operationalize them for your project.
    a. A full list of Bunger et al. strategies can be found at https://link.springer.com/article/10.1186/s12961-017-0175-y.
    b. A full list of the Powell et al. (a.k.a. ERIC) strategies can be found at https://implementationscience.biomedcentral.com/articles/10.1186/s13012-015-0295-0/tables/1.
3. Add your discrete strategies to your logic model. See the completed logic model for examples. E.g., the PrEP example project used *education* (Bunger)/ *train and educate stakeholders* (ERIC) to train providers/staff on PrEP efficacy, eligibility, stigma, etc.

√	Strategy category	Example discrete strategies
	**Bunger et al., 2017; Powell, et al., 2012**	
	Planning	- Tailor strategies<br>- Identify and prep champions<br>- Develop blueprint<br>- Build buy-in<br>- Assess readiness, identify barriers
	Education	- Informal local opinion leaders<br>- Conduct educational meetings<br>- Distribute materials<br>- Conduct ongoing training
	Finance	- Fund/contract<br>- Access new funding
	Restructure	- Change records systems<br>- Change structure/equipment<br>- Revise roles
	Quality management	- Develop systems<br>- Use data experts<br>- Clinical supervision<br>- Reminders<br>- Obtain worker feedback
	Policy	- Change policy context

	Expert Recommendations for Implementing Change (ERIC; Powell et al., 2015; Waltz et al., 2015)	
	Use evaluative and iterative strategies	- Assess for readiness and identify barriers and facilitators<br>- Audit and provide feedback<br>- Develop and implement tools for quality monitoring<br>- Conduct local need assessment<br>- Obtain and use patients/consumers and family feedback
	Provide interactive assistance	- Facilitation<br>- Provide local technical assistance<br>- Provide clinical supervision<br>- Centralize technical assistance
	Adapt and tailor to context	- Tailor strategies<br>- Promote adaptability<br>- Use data experts<br>- Use data warehousing techniques
	Develop stakeholder interrelationships	- Identify and prepare champions<br>- Organize clinician implementation team meetings<br>- Recruit, designate, and train for leadership<br>- Inform local opinion leaders<br>- Build a coalition<br>- Obtain formal commitments
	Train and educate stakeholders	- Conduct ongoing training<br>- Provide ongoing consultation<br>- Develop educational materials<br>- Distribute educational materials<br>- Use train-the-trainer strategies<br>- Create a learning collaborative
	Support clinicians	- Facilitate relay of clinical data to providers<br>- Remind clinicians<br>- Develop resource sharing agreements<br>- Revise professional roles<br>- Create new clinical teams
	Engage consumers	- Involve patients/consumers and family members<br>- Intervene with patients/consumers to enhance uptake and adherence<br>- Prepare patients/consumers to be active participants<br>- Increase demand<br>- Use mass media
	Utilize financial strategies	- Fund and contract for the clinical innovation<br>- Access new funding<br>- Alter incentive/allowance structures<br>- Make billing easier<br>- Alter patient/consumer fees
	Change infrastructure	- Mandate change<br>- Change record systems<br>- Change physical structure and equipment<br>- Change service sites
